# Book Reviews

**Published:** 1898-11

**Authors:** 


Transactions of the Medical Society of the State of New York. For the year 1898. Edited by Frederic C. Curtis, M. D., Secretary, Albany, N. Y. Octavo, pp. viii.—512. Philadelphia: Wm. J. Dornan, Printer. 1898.

This excellent medical society again presents its annual volume of transactions in attractive form. The book before us records the proceedings, scientific papers, discussions and other important matters pertaining to the 92d annual meeting of the society. Though one of the oldest medical organisations in the country it keeps itself ever young through the influence of new and enterprising members added each year, and by a spirit of general aggressiveness and progressiveness that is commendable

The form and method of publication adopted by this society is of the best and deserves to be imitated by other state societies, that are less careful in the publication of their transactions. The make-up of the book is due to two essential factors—an experienced secretary and a most excellent printer. Both should be kept as long as they will serve.

---

Atlas of Syphilis and the Venereal Diseases, Including a Brief Treatise on the Pathology and Treatment. By Prof. Dr. Franz Mracek, of Vienna. Authorised translation from the German. Edited by L. Bolton Bangs, M. D., Consulting Surgeon to St. Luke's Hospital and the City Hospital, New York, etc., etc. Duodecimo, pp. 122. With 71 colored plates. Philadelphia: W. B. Saunders. 1898.

This work is constructed upon lines that will aid the general practitioner of medicine amazingly in interpreting the various phenomena that follow syphilitic infection. The illustrations are numerous and clear, they are faithful depictions of clinical facts, and cannot fail to elucidate many hitherto obscure conditions, such as only the specialist of great skill ordinarily would be competent to explain. The characteristic thoroughness of the German is everywhere to be noted in the text as well as the plates. The diffusion of such literature amongst the profession of medicine is to be commended and especially upon the subject of which this book treats, on which there has, until somewhat lately, been so much taught that was not accurate, thus necessitating unlearning the false to make way for the true.

The editor has performed a valuable service in reducing the text to smooth English, without in any way modifying or perverting the author's ideas.

The book is one of vast possibilities for good and should be found in every physician's library.

---

DISEASES OF THE STOMACH. A Text-book for Practitioners and Students. By MAX EINHORN, M. D., Adjunct Professor in Clinical Medicine at the New York Post-Graduate Medical School and Hospital; Visiting Physician to the German Dispensary. Pp. 486. New York: William Wood & Co. Second revised edition. 1894.

Soon after the appearance of the first edition of this work, that is to say, in the issue of the JOURNAL for April, 1897, we published a somewhat extended review of it, and commended it to the profession of medicine in complimentary terms. That a new edition is called for so soon speaks importantly of its reception. Very few authors find their first editions exhausted within a year of their publication. Einhorn has taken advantage of this fact to make some alterations and a few additions to the text of his book, but there are no very radical changes, because there have been very few new principles added to the subject. An extended review is, therefore, uncalled for at this time, and we shall content ourselves with reminding our readers that this treatise was the first original work published in this country on the subject, and to say that it may be accepted as a safe guide by physicians as well as an excellent book for students.

---

A MANUAL OF MODERN SURGERY, GENERAL AND OPERATIVE. By JOHN CHALMERS DACOSTA, M. D., Clinical Professor of Surgery, Jefferson Medical College, Philadelphia, etc. Octavo, pp. 911. With 386 illustrations. Philadelphia: W. B. Saunders, 925 Walnut street. 1898.

The first edition of this work appeared in the Autumn of 1894 and in the issue of the JOURNAL for February, 1895, we expressed a favorable opinion of the book. The author announced in his first edition that it sought to stand between the text-book and the compend—a place that it most admirably fills. He has not changed the general scope or plan of the work in this edition, though he has practically rewritten the book. Many new articles have been added, and most of the old sections have been "enlarged, restricted or otherwise altered."

DaCosta does not discuss the specialties—ophthalmology, gynecology, laryngology and the like—but leaves them to the consideration of those trained in these lines. This, in our view, is wise, and it bespeaks a modesty on the part of this author not usually found among general surgeons. They generally wish to cover the whole field whether in practice or authorship, and often make sorry work of it.

One of the advantages of this work for the student will be found in the fact that obsolete and unessential methods have been left untouched; therefore he will not be confused in determining the best course to pursue in a given condition. The author devotes much space to the practical, and, per contra, but little to the theoretical, thus further enhancing the value of the book for the student and young practitioner of surgery. For example, extended space and comment are given to the consideration of the all-important subjects of fractures and dislocations—a praiseworthy fact.

By and large this work in material, illustration and mechanical make-up is in our opinion the best *manual* on surgery that has yet issued from the American press.

---

MANUAL OF PRACTICAL DIAGNOSIS FOR THE USE OF STUDENTS AND PHYSICIANS. By JAMES TYSON, M. D., Professor of Clinical Medicine in the University of Pennsylvania, and Physician to the University Hospital, etc. Small 8vo, pp. xii.—278. Third edition, revised and enlarged. Illustrated. Philadelphia: P. Blakiston, Son & Co., 1012 Walnut street. 1898.

It is always a genuine pleasure to take up a book that Tyson has written, for in it he is sure to say something new, even upon an old subject, or to say it in a new way, which is almost the same thing. This little work is distinctive in the direction named; in it will be found many ideas, or additions to principles, not found elsewhere. The story, too, is told in an entertaining way. This third edition of an important manual on a growingly important subject will be found useful to both physicians and students, though chiefly designed for the latter. Professor Tyson has made some changes and additions here not found in previous editions, and, altogether it is one of the most useful of all the small medical books published.

---

ATLAS AND ABSTRACT OF THE DISEASES OF THE LARYNX. By L. GRÜNWALD, M. D., of Munich. Authorised translation from the German. Edited by CHARLES P. GRAYSON, M. D., Lecturer on Laryngology in the University of Pennsylvania, etc., etc. With 107 colored figures and 44 plates. Philadelphia: W. B. Saunders. 1898.

The object of this book, as stated by its author, is to help the beginners in the art of observing and interpreting. The study of diseases of the larynx has always been attended with extreme difficulty, especially in the acute stages. It is true the laryngoscope of late has simplified the matter somewhat, yet only a few are prepared to give a pathologic description of what they see, or even make a mental picture of the field sufficiently accurate to guide in treatment. This work will aid vastly in clearing up doubt and may well be studied by specialists as well as students and practitioners. The pictures are beautiful works of art and are noted for faithfully depicting just what they are intended to elucidate. Illustrations do not always do this, so we desire to emphasise this point in the work. It deserves a large sale and doubtless will obtain its deserts in this respect.

LECTURES ON TUMORS. By JOHN B. HAMILTON, M. D, LL. D., Professor of Surgery, Rush Medical College and Chicago Polyclinic; Surgeon to Presbyterian Hospital, etc., etc. Third edition. Illustrated. Philadelphia: P. Blakiston's Son & Co. 1898.

The importance of a thorough knowledge of the pathology, diagnosis and treatment of tumors is second to no other subject in surgery. These lectures are given by a masterful surgeon, who knows his subject and appreciates its importance. He makes it interesting by simplicity of speech and method of dealing with it. No student can fail to know something of tumors who will carefully study this monograph.

---

THE OFFICE TREATMENT OF HEMORRHOIDS, FISTULA, ETC., WITHOUT OPERATION. Together with Remarks on the Relation of Diseases of the Rectum to Other Diseases in Both Sexes, but Especially in Women, and the Abuse of the Operation of Colostomy. By CHARLES B. KELSEY, A. M., M. D., late Professor of Surgery at the New York Post-graduate Medical School and Hospital; Fellow of the New York Academy of Medicine, the New York County Medical Society, etc. Duodecimo, pp. 68. New York: E. R. Pelton, Publisher, No. 19 East Sixteenth street. 1898.

The foregoing is a large title to a small book. The author says that in presenting the three lectures contained in the book in this form, he has done what he could to counteract the far too prevalent idea in the medical profession, that the only radical treatment of these affections lies in operation, and to further call attention to the too frequent misuse of operative procedures. If he succeeds in this laudable endeavor he will do well; at all events the little brochure affords an hour's pleasant reading.

A Treatise on the Principles and Practice of Gynecology. By E. C. Dudley, A. M., M. D., Professor of Gynecology in the Chicago Medical College, Chicago. Octavo, pp. 632, with 422 engravings, of which 47 are in colors, and 2 colored plates. New York and Philadelphia: Lea Brothers & Co. 1898.

If it is true, as has been asserted, that there are too many text-books on gynecology, it is equally true that there are not too many good ones. We could wish that all deserved the high place in the literature of medicine that this work of Dudley merits. In the first place, it is written by a careful, conscientious observer, who well understands his subject and its needs. In the second place, it is limited to the presentation of only such material as experience has taught the author to regard as valuable, excluding everything not justified by pathology or observation as worthy of record.

Professor Dudley has made five grand divisions of his treatise, as follows: I. General Principles. II. Inflammations. III. Tumors, malformations and tubal pregnancy. IV. Traumatism. V. Displacements and pelvic massage. It will be observed that these follow pathological lines, rather than the usual plan of treating of the diseases of a special organ in separate chapters; in other words, it is primarily pathological and only secondarily regional in its arrangement. This is an excellent plan for students especially, because they are less apt to be confused by it than by the old way.

In the first part Dudley considers the physiological periods in the life of woman, which are five—namely, infancy, puberty, maturity, the menopause, and senility. Then he takes up in the next antiseptics, asepsis, diagnosis, local treatment, minor operations, major operations, drainage, after-treatment, and the relation of dress to the diseases of women. He demonstrates his practical knowledge in his preparation for operations and in the technique he describes. In the chapter on dress he has carefully pointed out an important factor in causing diseases of not only the abdominal and pelvic organs, but of the thoracic viscera as well. We wish every woman as well as every physician was properly impressed with the importance of this subject, and could know how she deforms and displaces her organs by the adoption of present methods of dress. The author might have enlarged the chapter very much, but what he has said is pointed and instructive.

Coming now to the second grand division, inflammations, we find the following order, infection, vulvitis and vaginitis, eczema, herpes, kraurosis, pruritis and hyperesthesia vulvæ, and vaginismus; inflammation of the uterus; acute metritis; chronic metritis; chronic endocervicitis; chronic myo-metritis; pelvic inflammation; pelvic cellulitis; inflammation of the uterine appendages—salpingitis, ovaritis, pelvic peritonitis; urethritis, prolapse of the urethra, sub-urethral abscess, cystitis, pyelitis. We have given the foregoing to show the order of sequence in which Dudley handles his subjects.

In the third part, in which tumors, tubal pregnancy and malformation are dealt with, our author is at his best. Here he gives us excellent pathology and admirable surgical technique. If now and then we would differ from him, it is only in relation to some minor point, and who shall say which of two methods is best when results are the same or similar? Too much dogmatism in medicine, especially in gynecology, is to be deplored. That method is best for an operator with which he is most familiar, and which enables him to make the best economy of time and shorten anesthesia.

In the fourth and fifth parts will be found much that is helpful to the physician who does not practise operative gynecology, as related to the abdomen and pelvis. Every gynecologist ought to be prepared to close an uncomplicated lacerated cervix or perineum and to treat ordinary displacements.

The final chapter is allotted to the consideration of pelvic massage, after the method of Brandt. It is illustrated with nineteen drawings, giving the various methods of its application. There is no question but that this is a valuable aid in appropriate cases, and that it has not yet been given the place it merits among gynecological resources. Its liability to misuse and its employment by professional fakirs has made physicians mistrustful of it and prevented its employment in many instances, but Dudley hopes what he has said of it will lead to its more definite recognition and scientific use. We trust these expectations may be fulfilled.

This treatise is quite elaborately illustrated with wood cuts, a few photographs, two colored plates, and a number of colored drawings. Some of these pictures are excellent, a few of them are antiquated, and the remainder seem to elucidate the text with reasonable clearness. The colors, illustrating congenital gynatresia, are especially to be commended. No one could fail to understand the pathology of this condition after a study of them. We wish we could give a careful analytical review of the work, but this neither time or space will permit. We, have, however, attempted to outline its scope and method, which is perhaps even better for our readers, for it will enable them to judge as to the proprietary of possessing it, and this is, after all, the most important question to be elaborated here. As a final word, however, we may truthfully add that no man, young or old, student or specialist, family doctor or consultant, will regret the time spent in the reading of this book as a whole, or in any special part. It is the kind of a treatise that the profession is better for having at hand.

---

PROCEEDINGS of the Tenth Annual Session of the Association of American Anatomists. Held at Cornell University, Ithaca, December 28-30, 1897. D. S. Lamb, M. D., Secretary.

This brochure of 142 pages cannot fail to interest everyone who is engaged in the teaching or study of anatomy. It was appropriate that the tenth session of the association should have been held at Ithaca, the home of Professor Wilder, a founder, and it was still more felicitous that the association shculd elect the distinguished anatomist and physiologist of Cornell University, to be its next president. The pamphlet contains a portrait of the late Dr. Harrison Allen as a frontispiece.

---

A GUIDE TO THE CLINICAL EXAMINATION OF THE BLOOD FOR DIAGNOSTIC PURPOSES. By RICHARD CABOT, M. D. With colored plates and engravings. Third revised edition. Octavo, pp. 462. New York: William Wood & Co. 1898.

The early appearance of the third edition of this most important and excellent work speaks volumes for its worth. It treats upon a comparatively new subject, and is the only volume in English yet published upon hematology. The author is one of the first scientists in this country to study this department of clinical medicine, and to him the profession is indebted for what little it has acquired in regard to it. We say " what little " understandingly, for there is so much yet apparently to be unfolded that the information already acquired seems but little indeed.

We have, however, already learned that it will not simply answer to pronounce a patient anemic and proceed to administer iron without a complete investigation, including at least a blood count. But we are now just concerned with the present volume and proceed to point out the additions or changes that have been made since a former edition was set forth.

The principal additions include an account of Oliver's tintometer and hemoglobinometer, new matter in the chapter on the primary anemias and on leukemia, and a description of Muller's "blood-dust" (the newly discovered constituent of normal and abnormal blood), 1,700 additional blood examinations, new observations on poisoning, on aneurisms, or cretinism, etc. The general plan of the book remains unchanged.

In a review of a former edition the JOURNAL entered into a detailed notice of the several books and parts into which the author divides his subject; but this is now not requisite. What the physician needs is to purchase the book at once, for no intelligent professional work can be done without an understanding of the principles and methods therein set forth. The plates illustrating the blood corpuscles are works of art and the whole make-up of the book is beyond criticism.

---

REPORT of the Commissioner of Education for the Year 1896-97. Volume I. Containing Part I. Washington: Government Printing Office. 1898.

These reports, always valuable, grow more and more so with each year. The present one deals largely with educational questions in Europe, which merit examination and study in comparison with our own system. The commissioner makes appropriate reference to General Francis A. Walker's educational work and recommends that the various papers he wrote be collected into a volume by themselves, remarking that such a book would take a place at once in the most select list of educational classics. We hope the department will adopt the commissioner's suggestion. The commissioner dwells upon the importance of art in education and makes many felicitous observations upon this most important subject. We certainly hope what he says will lead to a more direct attention to this question. All educators should obtain this report, and read carefully the many admirable features it contains and discusses.

